# Janus kinase inhibitor use and incident dry eye disease in rheumatoid arthritis: a real-world cohort study

**DOI:** 10.3389/fimmu.2026.1871716

**Published:** 2026-07-08

**Authors:** Ching-Chieh Lin, Yang-Chi Lin, Renin Chang, Chun-Yu Lin, Jiunn-Liang Chen

**Affiliations:** 1 Department of Medical Education and Research, Kaohsiung Veterans General Hospital, Kaohsiung, Taiwan; 2 School of Medicine, Chung Shan Medical University, Taichung, Taiwan; 3 Department of Medical Education, Chung Shan Medical University Hospital, Taichung, Taiwan; 4 Department of Recreation and Sports Management, Tajen University, Pingtung, Taiwan; 5 Department of Emergency Medicine, Kaohsiung Veterans General Hospital, Kaohsiung, Taiwan; 6 Division of Allergy, Immunology, and Rheumatology, Department of Internal Medicine, Kaohsiung Veterans General Hospital, Kaohsiung, Taiwan; 7 School of Medicine, National Yang Ming Chiao Tung University, Taipei, Taiwan; 8 Ophthalmology Department, Kaohsiung Veterans General Hospital, Kaohsiung, Taiwan; 9 Optometry Department, Shu-Zen Junior College of Medicine and Management, Kaohsiung, Taiwan

**Keywords:** anti-inflammatory agent, dry eye disease, janus kinase inhibitor, ocular surface, rheumatoid arthritis

## Abstract

**Purpose:**

To examine the association between systemic Janus kinase (JAK) inhibitor use and the incidence of newly diagnosed dry eye disease (DED) in patients with rheumatoid arthritis (RA).

**Methods:**

This multi-institutional retrospective cohort study used de-identified electronic health records from the United States Collaborative Network. Adults aged 18 years or older with RA were categorized according to systemic JAK inhibitor use or non-use. A secondary active-comparator analysis compared JAK inhibitor users with rituximab-treated patients. Propensity score matching was performed to balance demographic characteristics, comorbidities, medication history, ocular conditions, and healthcare utilization. The primary outcome was newly diagnosed DED. Time-to-event analyses were conducted using Kaplan–Meier methods and Cox proportional hazards models.

**Results:**

A total of 25,149 JAK inhibitor users and 150,250 non-users with RA were identified. After matching, each cohort contained 23,343 patients with balanced baseline characteristics. JAK inhibitor use was associated with a lower incidence of newly diagnosed DED compared with non-use within 1 year of follow-up (hazard ratio [HR], 0.633; 95% CI, 0.545–0.736). In the secondary active-comparator analysis, 6,637 JAK inhibitor users were matched to 6,637 rituximab-treated patients, and JAK inhibitor use remained associated with a lower incidence of newly diagnosed DED (HR, 0.613; 95% CI, 0.476–0.791). Individual-agent analyses showed directionally consistent associations for tofacitinib and upadacitinib. Findings were consistent across sensitivity analyses using varying follow-up durations and landmark definitions.

**Conclusion:**

In this large real-world cohort study, JAK inhibitor use was associated with a lower incidence of newly diagnosed DED among patients with RA, with similar findings in a rituximab active-comparator analysis. However, the observational design, residual confounding, and coding-based outcome definition preclude causal inference. Prospective studies incorporating standardized ocular surface assessments and direct RA disease activity measures are needed to clarify the clinical relationship between systemic JAK inhibition and DED.

## Introduction

Dry eye disease (DED) is a multifactorial ocular inflammatory disease, characterized by discomfort, foreign body sensation, visual disturbances, and tear instability. It not only makes the ocular surface more susceptible to damage but can also affect daily activities (1). As the most common ocular complication of rheumatoid arthritis (RA), DED reportedly affects 15% to 90% of the population, influencing both physical and mental well-being (2–4).

Over the past decade RA treatment has evolved considerably, including the introduction of Janus kinase (JAK) inhibitors as targeted synthetic disease-modifying antirheumatic drugs (tsDMARDs). Three JAK inhibitors, tofacitinib, upadacitinib, and baricitinib, are approved by the US Food and Drug Administration (FDA) for RA. The JAK/Signal transducer and activator of transcription (STAT) pathway plays a critical role in immune and inflammatory regulation, tissue repair, and hematopoiesis. Previous studies also demonstrated that aberrant JAK/STAT activation contributed to the pathogenesis of DED (5).

Recent evidence has extended the potential role of JAK inhibition beyond systemic autoimmune disease to immune-mediated ocular inflammation and ocular surface disease. A recent systematic review and meta-analysis reported that JAK inhibitors may be effective in controlling relapses of noninfectious inflammatory ocular diseases, including uveitis and scleritis (6). In addition, a prospective phase 1/2 study of patients with ocular chronic graft-versus-host disease suggested that systemic baricitinib may improve selected ocular surface outcomes in ocular chronic graft-versus-host disease (oGVHD) (7). These emerging data support the clinical plausibility that systemic JAK inhibition could influence ocular surface inflammation, although they do not directly establish its role in preventing dry eye disease in rheumatoid arthritis.

Given the systemic immunomodulatory effects of these agents and the potential role of JAK/STAT signaling in ocular surface inflammation and DED pathophysiology, JAK inhibitor use was hypothesized to be associated with a lower incidence of newly diagnosed DED among patients with RA. This large real-world retrospective cohort study therefore used data from the United States to examine the association between JAK inhibitor use and incident DED among patients with RA.

## Methods

### Data collection

This multi-institutional retrospective cohort study used de-identified electronic health record data from the US Collaborative Network of the TriNetX platform, which aggregates data from more than 116 million patients across approximately 70 healthcare organizations. All patient data were de-identified by TriNetX in accordance with Section §164.514(a) of the HIPAA Privacy Rule; accordingly, institutional review board approval was not required (8). The study adhered to the tenets of the Declaration of Helsinki and is reported in accordance with the STROBE guideline for cohort studies. Records were identified using International Classification of Diseases, Tenth Revision, Clinical Modification (ICD-10-CM) diagnosis codes, RxNorm medication codes, and the Anatomical Therapeutic Chemical classification, with medication harmonization performed by the TriNetX platform (9). The eligibility window spanned January 1, 2016, to December 31, 2025, and data were extracted on May 31, 2026.

### Study population

**Figure 1:**
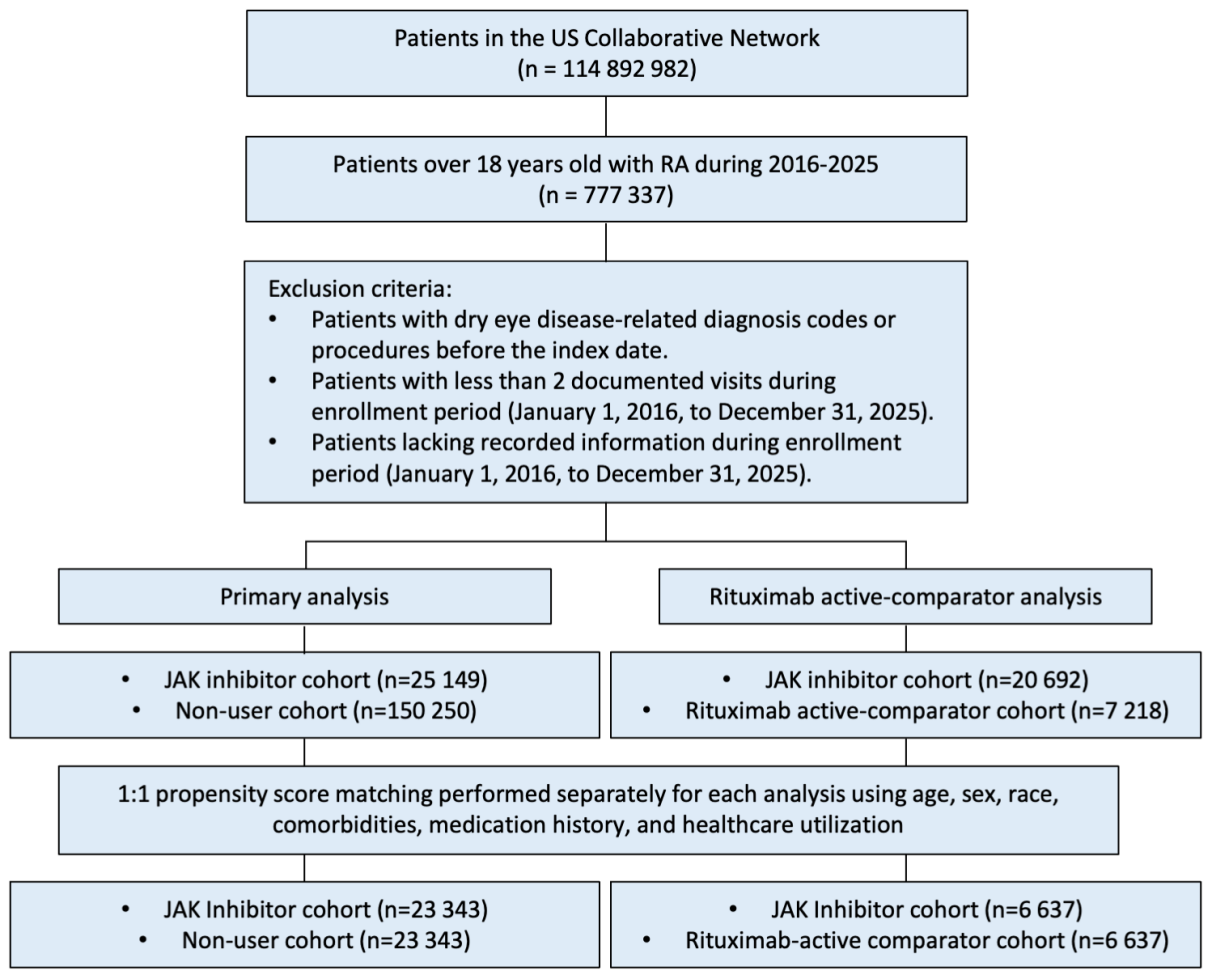
Study flowchart. The flowchart showing the construction of the study cohorts from the US Collaborative Network. Patients aged older than 18 years with rheumatoid arthritis during 2016–2025 were identified. Patients were excluded if they had dry eye disease-related diagnoses or procedures before the index date, fewer than 2 documented visits during the enrollment period, or missing recorded information during the enrollment period. Eligible patients were then assigned to the primary analysis comparing JAK inhibitor users with non-users and to the rituximab active-comparator analysis comparing JAK inhibitor users with rituximab users. In both analyses, 1:1 propensity score matching was performed using demographic characteristics, comorbidities, medication history, and healthcare utilization variables. Abbreviation: JAK, Janus kinase; RA, rheumatoid arthritis.

Adults aged ≥18 years with a diagnosis of RA (ICD-10-CM M05–M06) were eligible. To ensure data reliability, individuals with fewer than two documented visits in the database and those who lacked recorded information during the enrollment period from January 1, 2016, to December 31, 2025 were excluded. Patients with prior records of dry eye syndrome (ICD-10: H04.12), keratoconjunctivitis sicca not specified as Sjögren’s (ICD-10: H16.223 or H16.229), meibomian gland dysfunction (ICD-10 H02.88), lacrimal punctum closure by interventional management (CPT: 68760 or 68761), or Sjögren syndrome (ICD-10: M35.0) were also excluded. The flowchart for cohort construction is illustrated in **Figure 1**.

Three RA cohorts were defined. The JAK inhibitor cohort comprised patients with at least one prescription for tofacitinib, upadacitinib, or baricitinib. The non-user cohort comprised RA patients with a general-examination encounter and no record of JAK inhibitor use. The rituximab active-comparator cohort comprised RA patients with at least one rituximab prescription. For all cohorts, the first qualifying prescription or encounter had to occur on or after the first recorded RA diagnosis.

### Propensity score matching

To reduce baseline confounding, propensity score matching (PSM) was performed separately for each analysis and was estimated by logistic regression using covariates assessed during the 1 year before the index date, comprising demographic characteristics (age, sex, and race), RA-related medications, systemic comorbidities, ocular inflammatory condition and ophthalmologic healthcare utilization. The complete covariate list and codes are detailed in **Supplementary Table 1**.

Matching used 1:1 nearest-neighbor greedy matching with a caliper of 0.1 pooled standard deviations of the logit of the propensity score, as implemented by the TriNetX platform. Regarding missing data, patients lacking recorded information required for cohort assignment, index-date definition, or covariate assessment were excluded during cohort construction. Covariate balance before and after matching was assessed using the standardized mean difference (SMD), with an absolute SMD below 0.1 considered indicative of adequate balance (10).

### Outcome measurement

The investigated outcome was newly diagnosed DED, defined by ICD-10-CM codes H04.12, H16.223, H16.229, and M35.01, with a maximal follow-up of 5 years. Patients were followed from the index date until the first qualifying DED diagnosis, the end of available follow-up, or 5 years, whichever came first.

In the primary analysis, the risk of DED was compared between the JAK inhibitor cohort and the non-user cohort within a 1-year follow-up period, both before and after PSM. As secondary analyses, the rituximab active-comparator analysis compared the JAK inhibitor cohort with the rituximab active-comparator cohort using the same outcome definition, and the subgroup analysis stratified the JAK inhibitor cohort by specific agent, tofacitinib, upadacitinib, and baricitinib.

The index date was the date of this first qualifying prescription or encounter, so all patients entered as incident users of their respective treatment, and exposure was defined at treatment initiation following a first-exposure approach. For the rituximab active-comparator analysis, the two cohorts were further restricted to be mutually exclusive, with no prior exposure to the comparator agent, constituting a new-user, active-comparator design. For the individual-agent subgroup analysis, patients who switched between JAK inhibitors were excluded to reduce exposure misclassification. Data on dose, treatment duration, persistence, adherence, and discontinuation were unavailable and were not modeled. Cohort construction is summarized in **Figure 1**.

### Statistical analysis and sensitivity analysis

The cumulative incidence of DED was estimated using the Kaplan–Meier method and compared using the log-rank test. Hazard ratios (HRs) with 95% confidence intervals (CIs) were estimated using Cox proportional hazards models. The proportional hazards assumption was assessed using the TriNetX proportionality test based on scaled Schoenfeld residuals, as developed by Grambsch and Therneau. All tests were two-sided, with significance set at P <.05. Analyses and figures were generated in R version 4.4.1.

Two sets of sensitivity analyses assessed robustness. First, the follow-up duration analyses applied varying follow-up windows after matching, from 6 months to 1 year and at 2, 3, and 5 years after the index date, to evaluate consistency over time. Second, the landmark analyses using 1-month, 3-month, and 6-month landmarks excluded patients diagnosed with DED before each landmark, reducing the potential influence of immortal time and early ascertainment near the index date.

## Results

### Patient demographics

A total of 25,149 patients with RA receiving JAK inhibitors and 150,250 patients with RA in the non-user cohort were identified. Before PSM, the JAK inhibitor cohort was younger than the non-user cohort (mean age, 56.3 vs 60.7 years), and women accounted for 78.6% and 73.4% of the two cohorts, respectively. Several baseline comorbidities and RA-related medication exposures were imbalanced before matching, including hypertension, hyperlipidemia, and methotrexate, adalimumab, leflunomide, and etanercept use (**Table 1**).

**Table 1:** Baseline characteristics of the JAK inhibitor and non-user cohorts before and after propensity score matching.

Variables	Before PSM			After PSM		
	JAK Inhibitor (N = 25 149)	Non-user (N = 150 250)	SMD	JAK Inhibitor (N = 23 343)	Non-user (N = 23 343)	SMD
Age at index						
Mean ± SD	56.3 ± 13.3	60.7 ± 13.8	0.326	56.7 ± 13.2	56.7 ± 13.9	0.025
Sex, No. (%)						
Female	19768(78.6)	110292(73.4)	0.122	18239(78.1)	18264(78.2)	0.003
Race, No. (%)						
White	18450(73.4)	113444(75.5)	0.049	17106(73.3)	17046 (73)	0.006
Black or African American	3220(12.8)	21678(14.4)	0.047	3050(13.1)	2982(12.8)	0.009
Asian	751 (3)	3396(2.3)	0.045	681(2.9)	707 (3)	0.007
Healthcare utilization, No. (%)						
Persons encountering health services for examinations	9121(36.3)	68677(45.7)	0.193	8590(36.8)	8337(35.7)	0.023
Ophthalmological services for established patient	269(1.1)	2454(1.6)	0.049	253(1.1)	219(0.9)	0.015
Ophthalmological services for new patient	132(0.5)	943(0.6)	0.014	122(0.5)	114(0.5)	0.005
Contact Lens Services	12 (0)	129(0.1)	0.015	10(0)	17(0.1)	0.012
Comorbidities, No. (%)						
Hypertension	7322(29.1)	67387(44.9)	0.33	7018(30.1)	6853(29.4)	0.015
Hyperlipidemia	3694(14.7)	35549(23.7)	0.229	3543(15.2)	3449(14.8)	0.011
Chronic obstructive pulmonary disease	1352(5.4)	13084(8.7)	0.131	1305(5.6)	1279(5.5)	0.005
Chronic kidney disease	1125(4.5)	13374(8.9)	0.178	1099(4.7)	1063(4.6)	0.007
Heart failure	976(3.9)	10564(7)	0.139	954(4.1)	895(3.8)	0.013
Thyroid disorder	3151(12.5)	26353(17.5)	0.141	2987(12.8)	2935(12.6)	0.007
Type 2 diabetes mellitus	2867(11.4)	27354(18.2)	0.193	2737(11.7)	2672(11.4)	0.009
Psoriasis	1671(6.6)	5765(3.8)	0.126	1505(6.4)	1592(6.8)	0.015
Systemic lupus erythematosus	683(2.7)	4680(3.1)	0.024	644(2.8)	607(2.6)	0.01
Systemic involvement of connective tissue	349(1.4)	1658(1.1)	0.026	314(1.3)	277(1.2)	0.014
Sarcoidosis	117(0.5)	1067(0.7)	0.032	113(0.5)	102(0.4)	0.007
Conjunctivitis	177(0.7)	1625(1.1)	0.04	161(0.7)	130(0.6)	0.017
Iridocyclitis	117(0.5)	611(0.4)	0.009	108(0.5)	104(0.4)	0.003
Keratitis	29(0.1)	223(0.1)	0.009	28(0.1)	31(0.1)	0.004
Blepharitis	28(0.1)	352(0.2)	0.03	25(0.1)	32(0.1)	0.009
Herpes zoster infection	245(1)	1664(1.1)	0.013	231(1)	223(1)	0.003
Human immunodeficiency virus infection	17(0.1)	382(0.3)	0.047	17(0.1)	18(0.1)	0.002
Adenovirus infection	10(0)	10(0)	0.022	10(0)	0(0)	0.029
Acute hepatitis B	10(0)	55(0)	0.002	10(0)	11(0)	0.002
Chronic hepatitis	10(0)	81(0.1)	0.007	10(0)	10(0)	0
Tobacco use	534(2.1)	5055(3.4)	0.076	503(2.2)	485(2.1)	0.005
Alcohol related disorders	249(1)	2584(1.7)	0.063	239(1)	217(0.9)	0.01
Medications, No. (%)						
Steroids	2565(10.2)	10033(6.7)	0.127	2258(9.7)	2189(9.4)	0.01
Non-steroidal anti-inflammatory drugs	8495(33.8)	40396(26.9)	0.15	7658(32.8)	7307(31.3)	0.032
Methotrexate	7095(28.2)	16911(11.3)	0.436	6139(26.3)	6539(28)	0.039
Hydroxychloroquine	4175(16.6)	15421(10.3)	0.187	3696(15.8)	3673(15.7)	0.003
Adalimumab	3829(15.2)	4299(2.9)	0.441	2898(12.4)	2880(12.3)	0.002
Leflunomide	2888(11.5)	3878(2.6)	0.354	2187(9.4)	2210(9.5)	0.003
Etanercept	2922(11.6)	3341(2.2)	0.377	2173(9.3)	2132(9.1)	0.006
Sulfasalazine	1694(6.7)	3817(2.5)	0.2	1433(6.1)	1471(6.3)	0.007
Abatacept	1725(6.9)	1229(0.8)	0.318	1086(4.7)	979(4.2)	0.022
Tocilizumab	1029(4.1)	767(0.5)	0.241	669(2.9)	563(2.4)	0.028
Infliximab	514(2)	1418(0.9)	0.091	456(2)	447(1.9)	0.003
Certolizumab pegol	539(2.1)	445(0.3)	0.169	354(1.5)	325(1.4)	0.01
Rituximab	339(1.3)	1048(0.7)	0.065	305(1.3)	290(1.2)	0.006
Golimumab	497(2)	492(0.3)	0.155	349(1.5)	335(1.4)	0.005
Azathioprine	315(1.3)	1126(0.7)	0.051	285(1.2)	299(1.3)	0.005
Cyclosporine	174(0.7)	659(0.4)	0.034	151(0.6)	149(0.6)	0.001
Sarilumab	185(0.7)	96(0.1)	0.107	105(0.5)	84(0.4)	0.014
Anakinra	38(0.2)	103(0.1)	0.025	36(0.2)	28(0.1)	0.009

Data are presented as n (%) for categorical variables and mean ± SD for continuous variables. Covariates were assessed during the 1 year before the index date, and cohorts were matched 1:1 by nearest-neighbor propensity score matching; an absolute SMD < 0.1 indicated adequate balance.

JAK, Janus kinase; NSAIDs, non-steroidal anti-inflammatory drugs; PSM, propensity score matching; SD, standard deviation; SMD, standardized mean difference.

After PSM, 23,343 patients remained in each cohort. Baseline demographic characteristics, systemic comorbidities, ophthalmologic healthcare utilization, ocular inflammatory conditions, and medication history were well balanced, with all SMDs below 0.1 (**Table 1**; **Supplementary Figure 1**). The mean age was 56.7 years in both cohorts, and women accounted for 78.1% of the JAK inhibitor cohort and 78.2% of the non-user cohort. The most common systemic comorbidities after matching were hypertension, hyperlipidemia, thyroid disorder, and type 2 diabetes mellitus (**Table 1**).

### Risk of dry eye disease

**Figure 2:**
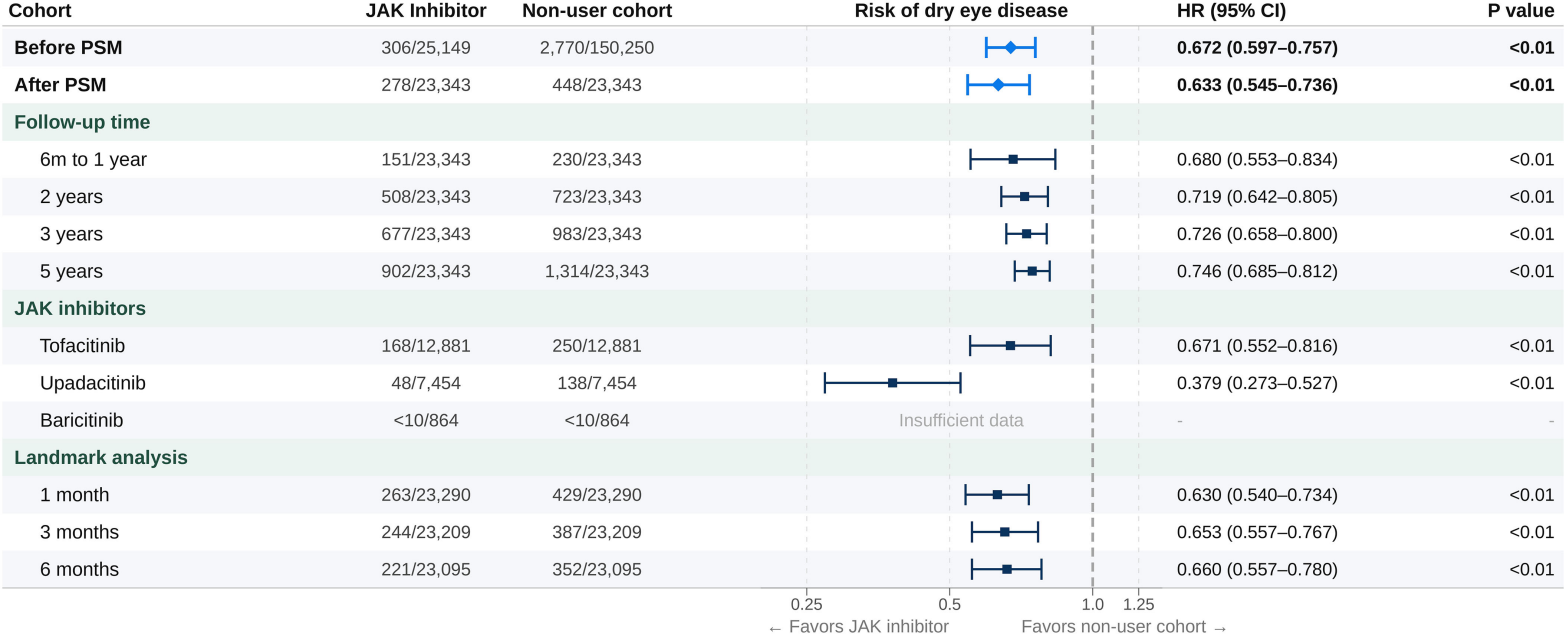
Risk of incident dry eye disease among patients with rheumatoid arthritis receiving JAK inhibitors compared with non-users. Forest plot showing the association between JAK inhibitor use and incident dry eye disease in the primary comparison with the non-user cohort. Hazard ratios were estimated before and after propensity score matching, across different follow-up durations, by individual JAK inhibitor agents, and in landmark analyses. Event counts are presented as the number of dry eye disease events divided by the total number of patients in each cohort. For baricitinib, HRs were not estimated because the number of events was insufficient for reliable analysis. Abbreviation: CI, confidence interval; HR, hazard ratio; JAK, Janus kinase; No., number; PSM, propensity score matching.

**Figure 3:**
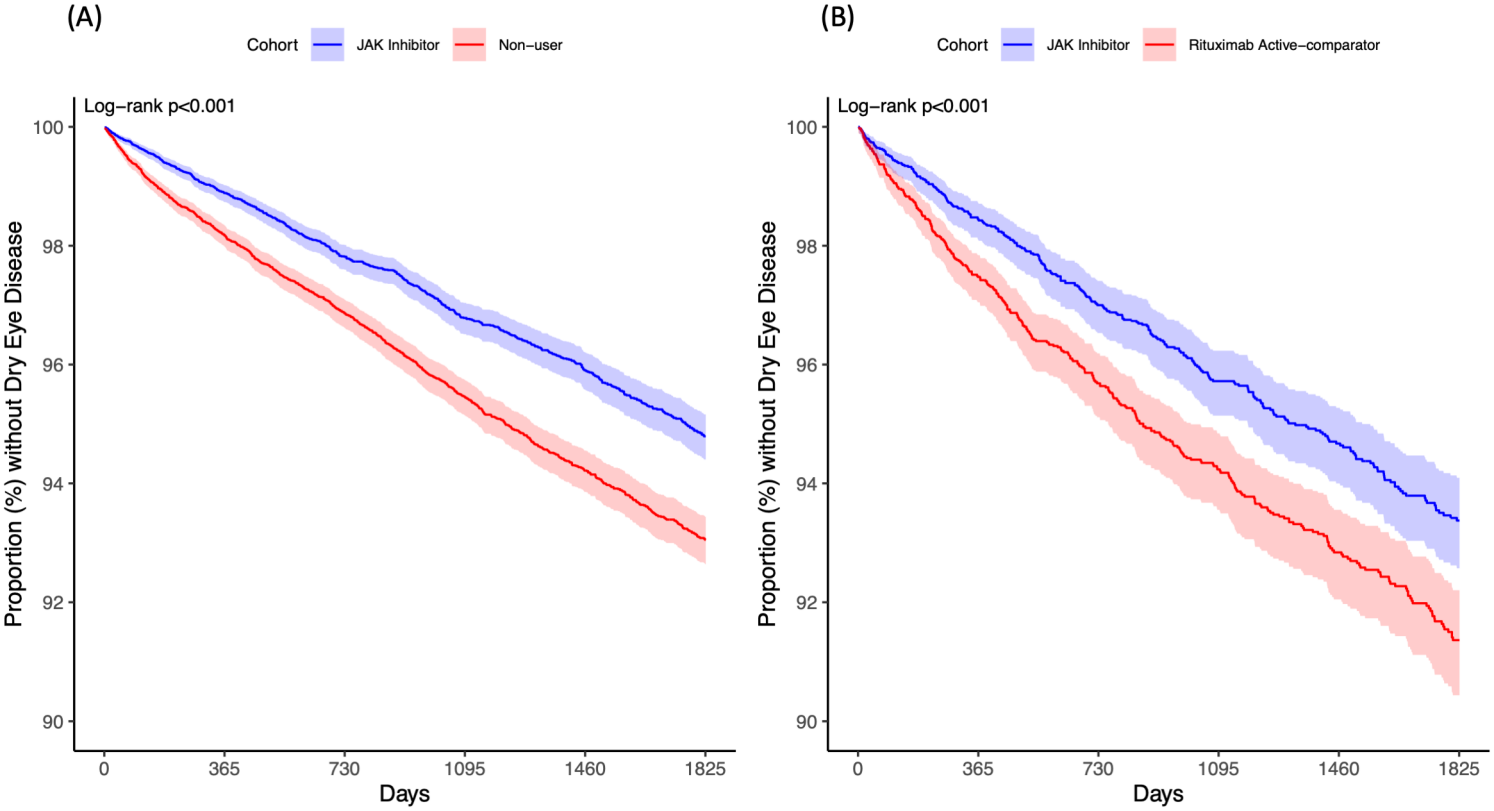
Kaplan–Meier plots of dry eye disease. Kaplan–Meier curves showing the proportion of patients remaining free from dry eye disease during follow-up. **(A)** compares the JAK inhibitor cohort with the non-user cohort. **(B)** compares the JAK inhibitor cohort with the rituximab active-comparator cohort. In both analyses, patients receiving JAK inhibitors showed a higher dry eye disease-free probability over time. Differences between curves were significant by log-rank test. The y-axis represents the proportion of patients without dry eye disease. The x-axis represents follow-up time in days. Shaded areas indicate 95% confidence intervals. Log-rank p < 0.001 for both comparisons. Abbreviation: JAK, Janus kinase.

In the primary non-user comparator analysis before PSM, newly diagnosed DED occurred in 306 of 25,149 patients in the JAK inhibitor cohort and 2,770 of 150,250 patients in the non-user cohort; JAK inhibitor use was associated with a lower incidence of newly diagnosed DED (HR, 0.672; 95% CI, 0.597–0.757; P <.01). After PSM, DED occurred in 278 of 23,343 patients in the JAK inhibitor cohort and 448 of 23,343 patients in the non-user cohort, and the association remained statistically significant (HR, 0.633; 95% CI, 0.545–0.736; P <.01), corresponding to an absolute risk difference of −0.73 percentage points. Kaplan–Meier analysis showed a correspondingly lower cumulative incidence of DED in the JAK inhibitor cohort than in the matched non-user cohort (log-rank P <.001) (**Figures 2**, **3**).

### Rituximab active-comparator analysis

**Figure 4:**
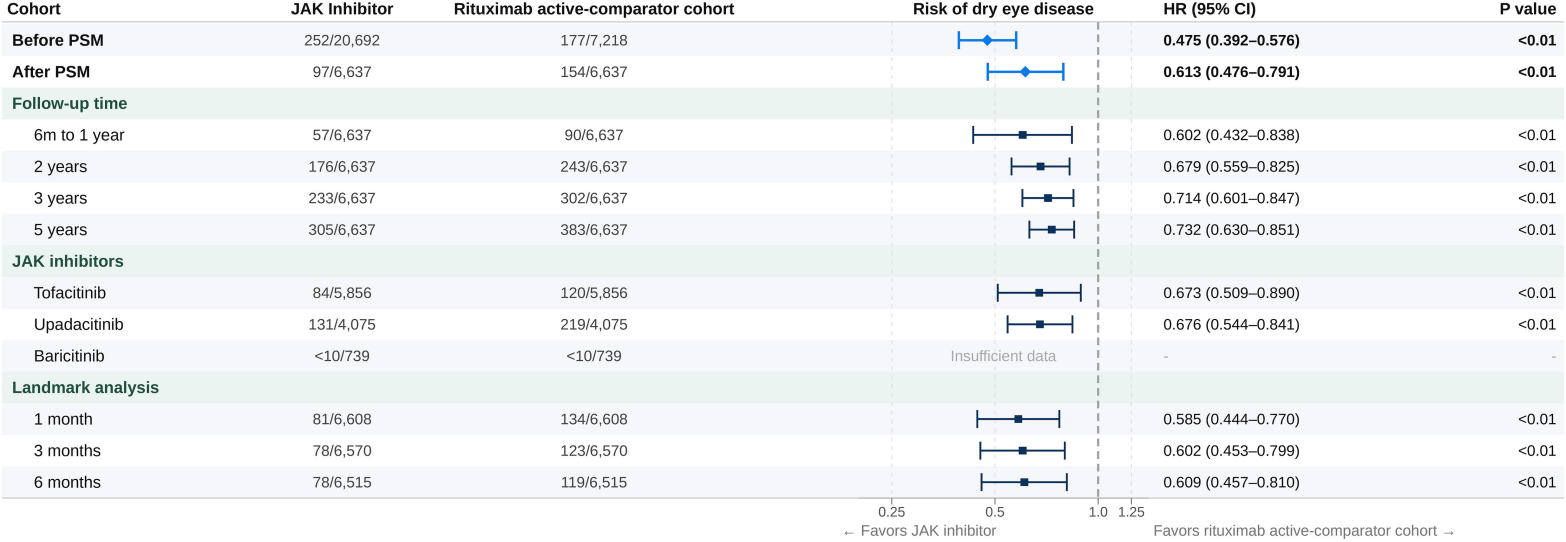
Risk of incident dry eye disease among patients with rheumatoid arthritis receiving JAK inhibitors compared with rituximab users. Forest plot showing the association between JAK inhibitor use and incident dry eye disease in the rituximab active-comparator analysis. Hazard ratios were estimated before and after propensity score matching, across different follow-up durations, by individual JAK inhibitor agents, and in landmark analyses. Event counts are presented as the number of dry eye disease events divided by the total number of patients in each cohort. For baricitinib, HRs were not estimated because the number of events was insufficient for reliable analysis. Abbreviation: CI, confidence interval; HR, hazard ratio; JAK, Janus kinase; No., number; PSM, propensity score matching.

To reduce confounding by indication, the rituximab active-comparator analysis compared the JAK inhibitor cohort with the rituximab active-comparator cohort. Before PSM, DED occurred in 252 of 20,692 patients in the JAK inhibitor cohort and 177 of 7,218 patients in the rituximab active-comparator cohort (HR, 0.475; 95% CI, 0.392–0.576; P <.01). After PSM, 6,637 patients remained in each cohort, and all covariates were balanced (all SMDs < 0.1; **Table 2**; **Supplementary Figure 2**). DED occurred in 97 of 6,637 patients in the JAK inhibitor cohort and 154 of 6,637 patients in the rituximab active-comparator cohort, and the association remained statistically significant (HR, 0.613; 95% CI, 0.476–0.791; P <.01), corresponding to an absolute risk difference of −0.86 percentage points. Kaplan–Meier analysis likewise showed a lower cumulative incidence of DED in the JAK inhibitor cohort than in the rituximab active-comparator cohort (log-rank P <.001) (**Figures 3**, **4**).

**Table 2:** Baseline characteristics of the JAK inhibitor and rituximab active-comparator cohorts before and after propensity score matching.

Variables	Before PSM			After PSM		
	JAK Inhibitor (N = 20 692)	Rituximab (N = 7 218)	SMD	JAK inhibitor (N = 6 637)	Rituximab (N = 6 637)	SMD
Age at index						
Mean ± SD	56.4 ± 13.2	59.7 ± 14.1	0.242	60.5 ± 12.8	59.9 ± 14	0.045
Sex, No. (%)						
Female	16283(78.7)	5232(72.5)	0.145	4907(73.9)	4844(73)	0.021
Race, No. (%)						
White	15257(73.7)	5016(69.5)	0.094	4739(71.4)	4699(70.8)	0.013
Black or African American	2661(12.9)	1127(15.6)	0.079	932(14)	961(14.5)	0.012
Asian	587(2.8)	170(2.4)	0.03	154(2.3)	157(2.4)	0.003
Healthcare utilization, No. (%)						
Persons encountering health services for examinations	7405(35.8)	3116(43.2)	0.151	2739(41.3)	2747(41.4)	0.002
Ophthalmological services for established patient	210(1)	123(1.7)	0.06	91(1.4)	95(1.4)	0.005
Ophthalmological services for new patient	107(0.5)	61(0.8)	0.04	48(0.7)	46(0.7)	0.004
Contact Lens Services	11(0.1)	10(0.1)	0.028	10(0.2)	10(0.2)	0
Comorbidities, No. (%)						
Hypertension	6084(29.4)	3011(41.7)	0.259	2611(39.3)	2607(39.3)	0.001
Hyperlipidemia	3063(14.8)	1567(21.7)	0.18	1392(21)	1366(20.6)	0.01
Chronic obstructive pulmonary disease	1170(5.7)	687(9.5)	0.146	614(9.3)	597(9)	0.009
Chronic kidney disease	950(4.6)	805(11.2)	0.245	631(9.5)	610(9.2)	0.011
Heart failure	814(3.9)	719(10)	0.239	561(8.5)	538(8.1)	0.013
Thyroid disorder	2587(12.5)	1249(17.3)	0.135	1096(16.5)	1087(16.4)	0.004
Type 2 diabetes mellitus	2384(11.5)	1133(15.7)	0.122	1012(15.2)	1008(15.2)	0.002
Psoriasis	1351(6.5)	174(2.4)	0.2	161(2.4)	170(2.6)	0.009
Systemic lupus erythematosus	511(2.5)	682(9.4)	0.298	423(6.4)	427(6.4)	0.002
Systemic involvement of connective tissue	264(1.3)	348(4.8)	0.207	178(2.7)	199(3)	0.019
Sarcoidosis	99(0.5)	97(1.3)	0.091	68(1)	72(1.1)	0.006
Conjunctivitis	139(0.7)	79(1.1)	0.045	72(1.1)	61(0.9)	0.017
Iridocyclitis	87(0.4)	48(0.7)	0.033	39(0.6)	37(0.6)	0.004
Keratitis	22(0.1)	50(0.7)	0.093	19(0.3)	27(0.4)	0.021
Blepharitis	21(0.1)	15(0.2)	0.027	10(0.2)	10(0.2)	0
Herpes zoster infection	203(1)	122(1.7)	0.062	93(1.4)	103(1.6)	0.012
Human immunodeficiency virus infection	14(0.1)	10(0.1)	0.022	10(0.2)	10(0.2)	0
Adenovirus infection	10(0)	10(0.1)	0.03	10(0.2)	10(0.2)	0
Acute hepatitis B	10(0)	10(0.1)	0.03	10(0.2)	10(0.2)	0
Chronic hepatitis	10(0)	10(0.1)	0.03	10(0.2)	10(0.2)	0
Tobacco use	460(2.2)	242(3.4)	0.069	229(3.5)	212(3.2)	0.014
Alcohol related disorders	200(1)	107(1.5)	0.047	94(1.4)	92(1.4)	0.003
Medications, No. (%)						
Steroids	2055(9.9)	1533(21.2)	0.316	1212(18.3)	1193(18)	0.007
Non-steroidal anti-inflammatory drugs	6958(33.6)	2388(33.1)	0.012	2173(32.7)	2159(32.5)	0.004
Methotrexate	5856(28.3)	1643(22.8)	0.127	1571(23.7)	1554(23.4)	0.006
Hydroxychloroquine	3480(16.8)	1450(20.1)	0.084	1215(18.3)	1229(18.5)	0.005
Adalimumab	3163(15.3)	323(4.5)	0.368	322(4.9)	321(4.8)	0.001
Leflunomide	2392(11.6)	593(8.2)	0.112	636(9.6)	575(8.7)	0.032
Etanercept	2411(11.7)	279(3.9)	0.294	277(4.2)	277(4.2)	0
Sulfasalazine	1423(6.9)	382(5.3)	0.066	387(5.8)	365(5.5)	0.014
Abatacept	1330(6.4)	334(4.6)	0.079	325(4.9)	321(4.8)	0.003
Tocilizumab	753(3.6)	230(3.2)	0.025	229(3.5)	210(3.2)	0.016
Infliximab	414(2)	176(2.4)	0.03	164(2.5)	164(2.5)	0
Certolizumab pegol	443(2.1)	44(0.6)	0.132	39(0.6)	44(0.7)	0.01
Golimumab	400(1.9)	96(1.3)	0.048	85(1.3)	95(1.4)	0.013
Azathioprine	246(1.2)	366(5.1)	0.224	193(2.9)	204(3.1)	0.01
Cyclosporine	145(0.7)	77(1.1)	0.039	64(1)	60(0.9)	0.006
Sarilumab	132(0.6)	27(0.4)	0.037	26(0.4)	26(0.4)	0
Anakinra	26(0.1)	17(0.2)	0.026	10(0.2)	12(0.2)	0.007

Data are presented as n (%) for categorical variables and mean ± SD for continuous variables. Covariates were assessed during the 1 year before the index date, and cohorts were matched 1:1 by nearest-neighbor propensity score matching; an absolute SMD < 0.1 indicated adequate balance.

JAK, Janus kinase; NSAIDs, non-steroidal anti-inflammatory drugs; PSM, propensity score matching; SD, standard deviation; SMD, standardized mean difference.

### Individual-agent subgroup analysis

In the matched non-user comparator analysis, tofacitinib and upadacitinib were each associated with a lower incidence of newly diagnosed DED. Among tofacitinib users, DED occurred in 168 of 12,881 patients versus 250 of 12,881 matched non-user comparators (HR, 0.671; 95% CI, 0.552–0.816; P <.01). Among upadacitinib users, DED occurred in 48 of 7,454 patients versus 138 of 7,454 matched non-user comparators (HR, 0.379; 95% CI, 0.273–0.527; P <.01). A baricitinib-specific estimate was not reported because fewer than 10 DED events occurred in each group, precluding reliable estimation.

In the rituximab active-comparator analysis, tofacitinib and upadacitinib showed directionally consistent associations. DED occurred in 84 of 5,856 tofacitinib users versus 120 of 5,856 matched rituximab comparators (HR, 0.673; 95% CI, 0.509–0.890; P <.01), and in 131 of 4,075 upadacitinib users versus 219 of 4,075 matched rituximab comparators (HR, 0.676; 95% CI, 0.544–0.841; P <.01). A baricitinib-specific comparison was not estimated because of insufficient event counts.

### Sensitivity analyses

Analyses using varying follow-up durations were consistent with the primary analysis. In the non-user comparator analysis, JAK inhibitor use was associated with a lower incidence of DED from 6 months to 1 year after the index date (HR, 0.680; 95% CI, 0.553–0.834; P <.01) and at 2 years (HR, 0.719; 95% CI, 0.642–0.805; P <.01), 3 years (HR, 0.726; 95% CI, 0.658–0.800; P <.01), and 5 years (HR, 0.746; 95% CI, 0.685–0.812; P <.01).

Comparable findings were observed in the rituximab active-comparator analysis: from 6 months to 1 year (HR, 0.602; 95% CI, 0.432–0.838; P <.01) and at 2 years (HR, 0.679; 95% CI, 0.559–0.825; P <.01), 3 years (HR, 0.714; 95% CI, 0.601–0.847; P <.01), and 5 years (HR, 0.732; 95% CI, 0.630–0.851; P <.01).

Landmark analyses further supported the robustness of the findings. In the non-user comparator analysis, the association remained statistically significant using 1-month, 3-month, and 6-month landmark definitions (HR, 0.630 [95% CI, 0.540–0.734]; 0.653 [95% CI, 0.557–0.767]; and 0.660 [95% CI, 0.557–0.780], respectively). In the rituximab active-comparator analysis, the corresponding estimates were 0.585 (95% CI, 0.444–0.770), 0.602 (95% CI, 0.453–0.799), and 0.609 (95% CI, 0.457–0.810).

## Discussion

This multi-institutional retrospective cohort study of 25,149 JAK inhibitor users and 150,250 non-user comparator, and 7,218 rituximab-treated active comparators with RA, evaluated the association between JAK inhibitor use and newly diagnosed DED. After PSM, JAK inhibitor use was associated with a lower 1-year incidence of newly diagnosed DED compared with both the non-user cohort and a rituximab active-comparator cohort with no prior JAK inhibitor use. In the individual-agent subgroup analysis, tofacitinib and upadacitinib showed directionally consistent associations. Sensitivity analyses across varying follow-up durations and landmark definitions were consistent, supporting the robustness of the association.

Given that DED is the most prevalent ocular surface disorder in RA patients, affecting 15% to 90% of the population, clarifying their association carries important clinical implications (3, 11, 12). Several studies have explored the potential relationship between RA disease severity or duration and the risk of developing DED, though the findings have been mixed. Fujita et al. reported that RA disease activity was positively correlated with dry eye severity only in RA patients with secondary Sjögren’s syndrome, but not in the overall RA population (3). Lai et al. demonstrated a significantly increased risk of DED among RA patients compared to matched controls in a large nationwide cohort, but did not specifically examine the effects of disease duration or severity (13). Abd-Allah et al. further found that disease activity score-28 (DAS-28) did not correlate with ocular dryness measures, whereas longer RA duration was associated with worse Schirmer and ocular staining scores (14). Prior clinical evidence also suggests that systemic RA treatment may influence ocular surface parameters, as Usuba et al. reported improvements in tear production, conjunctival impression cytology, and goblet cell count among RA patients receiving TNF inhibitors. Although this small TNF inhibitor-treated cohort cannot be directly extrapolated to JAK inhibitors, it supports the broader possibility that systemic inflammatory control may affect RA-associated ocular surface disease (15).

In RA, dry eye is commonly associated with immune-mediated lacrimal gland dysfunction, leading to reduced tear production and a pro-inflammatory ocular surface milieu characterized by upregulated cytokines (11, 16, 17). Recent studies have highlighted a role for JAK/STAT3 signaling in amplifying this immune cascade in DED, which shares immunopathological features with RA (5, 18, 19). Enhanced JAK/STAT3 signaling further exacerbates epithelial damage and goblet cell loss (5). It was hypothesized that by modulating JAK/STAT-driven cytokine release and immune cell recruitment, JAK inhibitors might reduce DED risk, offering an additional immunomodulatory benefit beyond joint disease control.

Although evidence supporting the use of JAK inhibitors for the treatment of DED remains limited, a phase 1/2 trial of topical tofacitinib demonstrated improvements in both signs and symptoms of DED, while an experimental murine study by Stevenson W. et al. showed that topical tofacitinib inhibited ocular surface inflammation in a murine model by reducing IL-1β and IL-6 levels (20, 21). Beyond topical treatment, preliminary studies in oGVHD, including a small retrospective case series of systemic ruxolitinib and a single-arm phase 1/2 pilot trial of oral baricitinib, reported improvements in selected ocular surface or symptom measures, although changes in tear production measured by Schirmer testing were inconsistent or non-significant (7, 22). Recent findings revealed elevated JAK/STAT signaling in minor salivary glands from patients with Sjögren’s syndrome, which ex vivo JAK inhibitor therapy was able to normalize, thereby underscoring a broader role for JAK inhibition in mitigating glandular dryness (23).

Together, these studies provide biological and clinical plausibility for the observed association, but they do not establish that systemic JAK inhibitors prevent RA-associated DED. Because DED is a chronic and multifactorial ocular surface disease, the lower incidence of newly diagnosed DED among JAK inhibitor users may have several explanations and should not be interpreted as evidence of a direct ocular effect. Potential explanations include modulation of the ocular surface inflammatory milieu, improved systemic inflammatory control, reduced overall RA disease burden, or differences in symptom expression, healthcare utilization, and clinical recognition.

To address potential confounding by treatment indication, a secondary active-comparator analysis was performed using rituximab-treated patients with RA, because both JAK inhibitors and rituximab are used in patients requiring advanced RA therapy after inadequate response to conventional treatment (24, 25). This analysis provided a clinically relevant treatment-based comparison and showed a similar association between JAK inhibitor use and a lower incidence of newly diagnosed DED. This finding may be biologically plausible because JAK inhibitors directly modulate cytokine signaling pathways implicated in ocular surface inflammation, whereas rituximab primarily targets CD20-positive B cells and has not shown consistent improvement in objective tear production or glandular function in primary Sjögren’s syndrome (26–28). However, these findings cannot be directly extrapolated to RA-associated DED, and no study has directly compared JAK inhibitors with rituximab for ocular surface outcomes. Thus, the rituximab active-comparator analysis should be interpreted as supportive but indirect evidence, given potential residual confounding from unmeasured RA severity, disease duration, prior treatment history, and inflammatory burden.

Sensitivity analyses using different follow-up windows and landmark definitions yielded consistent results, suggesting that the observed association was not limited to early post-index events or a single follow-up horizon. Although HRs were slightly attenuated with longer follow-up, the direction of association remained consistent, supporting the robustness of the findings.

### Strengths and limitations

This cohort study has several strengths. To our knowledge, it is the first large-scale real-world study to examine the association between systemic JAK inhibitor use and newly diagnosed DED among patients with RA, including a secondary active-comparator analysis using rituximab-treated patients. The large multi-institutional cohort provided real-world evidence across diverse healthcare settings. Propensity score matching was used to balance demographic factors, systemic comorbidities, ophthalmologic healthcare utilization, ocular inflammatory conditions, and RA-related medication history. The rituximab active-comparator analysis allowed comparison with a clinically relevant treatment-based cohort with potentially more similar disease severity and treatment indications than the general examination comparator cohort. In addition, a new-user design was used, with follow-up beginning at the first recorded prescription of the index medication, and patients with treatment switching between JAK inhibitors and rituximab were excluded to reduce exposure misclassification.

Subgroup analyses showed directionally consistent associations for tofacitinib and upadacitinib, whereas baricitinib-specific estimates could not be reliably assessed because of insufficient event counts. Sensitivity analyses using alternative follow-up durations and landmark definitions further supported the robustness of the findings.

Several limitations should be acknowledged. First, direct measures of RA disease activity, including DAS-28, inflammatory biomarkers, disease duration, seropositivity, functional status, and treatment response, were unavailable. Therefore, residual confounding by disease severity and treatment indication cannot be excluded, and the observed association may reflect improved systemic inflammatory control or differences in underlying RA severity rather than direct ocular effects. Second, although ophthalmologic healthcare utilization and ocular comorbidities were balanced after matching, residual surveillance bias related to specialist follow-up, care-seeking behavior, referral patterns, or diagnostic thresholds may remain.

Third, DED was identified using diagnostic codes rather than standardized clinical assessments, such as tear break-up time, Schirmer testing, corneal staining, tear osmolarity, or symptom questionnaires. ICD-based definitions were used because structured ophthalmologic examination data were not consistently available across participating healthcare organizations. Accordingly, this study evaluated newly diagnosed, healthcare-documented DED rather than changes in disease severity, symptom burden, tear production, or clinical improvement.

Finally, as an observational retrospective cohort study, causal inference cannot be established. Information on medication dosage, treatment duration, adherence, discontinuation, and reasons for treatment selection was unavailable. Prospective studies incorporating standardized ocular surface assessments, direct RA disease activity measures, and longitudinal treatment exposure data are needed to clarify the relationship between systemic JAK inhibition and DED in patients with RA.

In conclusion, this large real-world cohort study identified an association between JAK inhibitor use and a lower incidence of newly diagnosed DED among patients with RA, with similar findings in the rituximab active-comparator analysis. However, residual confounding and limitations inherent to retrospective database studies preclude causal inference.

## Data Availability

The datasets presented in this article are not readily available because the datasets used in this study were obtained from the TriNetX network, a federated electronic health record platform that provides access to de-identified patient data. Due to data use agreements and institutional policies, individual-level data cannot be shared or exported outside the platform. Access to the dataset is restricted to authorized users through the TriNetX system and is subject to compliance with data governance, privacy regulations, and institutional requirements. Researchers interested in accessing the data may do so through the TriNetX platform upon approval. Requests to access the datasets should be directed to Name: TriNetX Research Network Website: https://trinetx.com Access information: https://trinetx.com/contact-us/.
