# Supplementary material for: Janus kinase inhibitor use and incident dry eye disease in rheumatoid arthritis: a real-world cohort study
Source: Front Immunol. 2026 Jul 8;17:1871716. doi: 10.3389/fimmu.2026.1871716 (PMC13388308; doi:10.3389/fimmu.2026.1871716)
Supplement: Supplementary file 1 [file DataSheet1.pdf]

Supplementary Material

Supplementary Table S1. Covariates and corresponding ICD-10-CM codes used for propensity score matching

Supplementary Figure S1. Covariate balance before and after propensity score matching in the primary analysis.

Supplementary Figure S2. Covariate balance before and after propensity score matching in the rituximab active-comparator analysis.

Supplementary Table S1. Covariates and corresponding ICD-10-CM codes used for propensity score matching

Code	Comorbidities
Z72.0	Tobacco use
F10	Alcohol-related disorders
E11	Type 2 diabetes mellitus
I10–I1A	Hypertensive diseases
E78.5	Hyperlipidemia
N18	Chronic kidney disease
I50	Heart failure
J44	Chronic obstructive pulmonary disease
E00–E07	Disorders of the thyroid gland
D86	Sarcoidosis
M35.9	Systemic involvement of connective tissue
M32	Systemic lupus erythematosus
L40	Psoriasis
B20	Human immunodeficiency virus disease
B02	Herpes zoster infection
B34.0	Adenovirus infection
B16	Acute hepatitis B virus infection
Z79.5	Long-term (current) use of steroids
H16	Keratitis
H10	Conjunctivitis
H01.0	Blepharitis
H20	Iridocyclitis
Code	Medication
RxNorm 614391	Abatacept
RxNorm 327361	Adalimumab
RxNorm 72435	Anakinra
RxNorm 1256	Azathioprine
RxNorm 709271	certolizumab pegol

RxNorm 3008	Cyclosporine
RxNorm 214555	Etanercept
RxNorm 819300	Golimumab
RxNorm 5521	Hydroxychloroquine
RxNorm 191831	Infliximab
RxNorm 27169	Leflunomide
RxNorm 6851	Methotrexate
ATC M01A	Non-steroidal anti-inflammatory drugs
RxNorm 121191	Rituximab
RxNorm 1923319	Sarilumab
RxNorm 9524	Sulfasalazine
ICD-10 Z79.5	Steroids
RxNorm 612865	Tocilizumab
Code	Healthcare utilization
ICD-10 Z00-Z13	Persons encountering health services for examinations
CPT 92014	Ophthalmological services for established patient
CPT 92004	Ophthalmological services for new patient
CPT 1012841	Contact Lens Services

Abbreviation: ATC, Anatomical Therapeutic Chemical Classification System; CPT, Current Procedural Terminology; ICD-10-CM, International Classification of Diseases, Tenth Revision, Clinical Modification.

Supplementary Figure S1. Covariate balance before and after propensity score matching in the primary analysis.

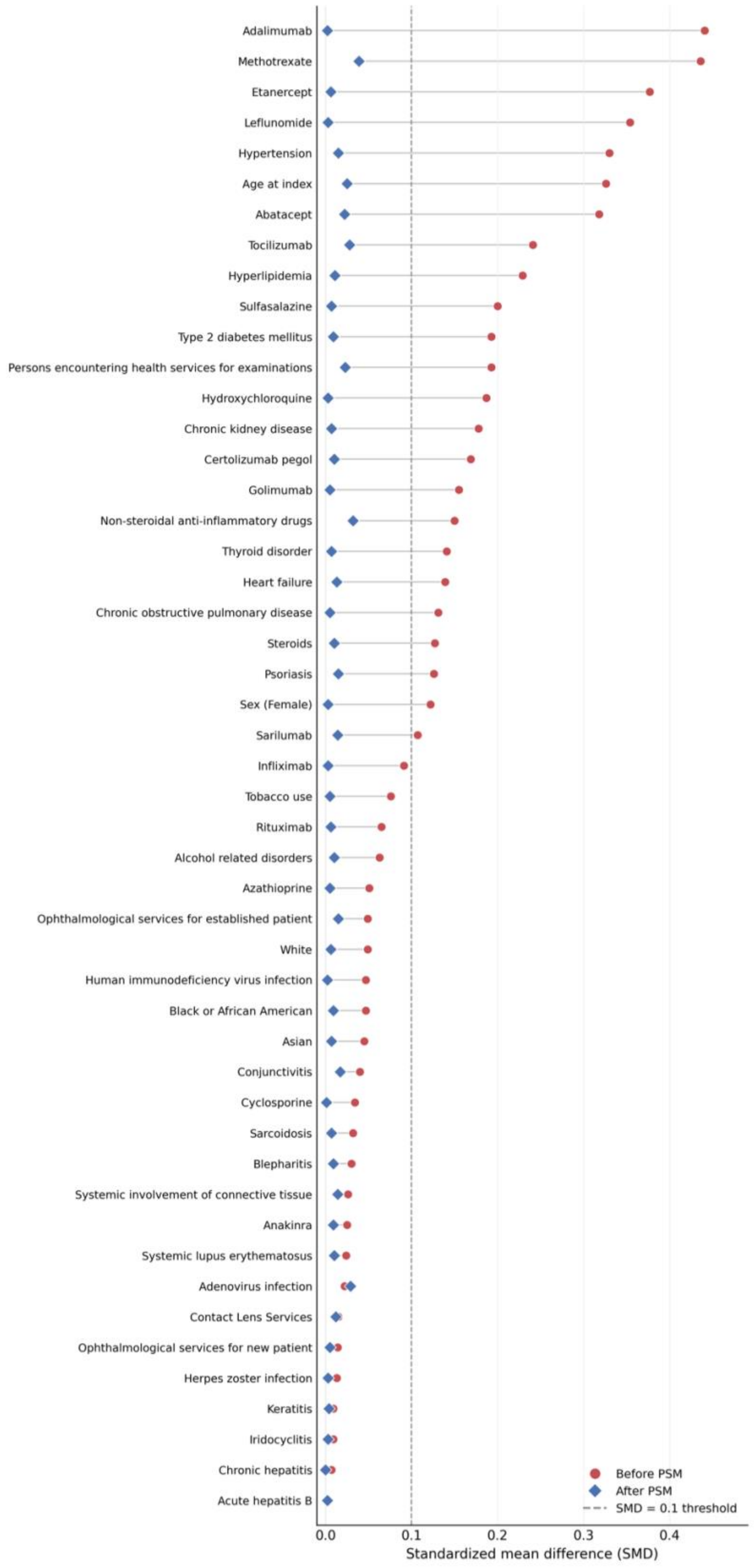

Love plot showing standardized mean differences for baseline covariates before and after propensity score matching between the JAK inhibitor cohort and the non-user cohort. After matching, all covariates were balanced with standardized mean differences below 0.1. Red circles represent covariate balance before PSM, and blue diamonds represent covariate balance after PSM.

Abbreviation: JAK, Janus kinase; PSM, propensity score matching; SMD, standardized mean difference.

Supplementary Figure S2. Covariate balance before and after propensity score matching in the rituximab active-comparator analysis.

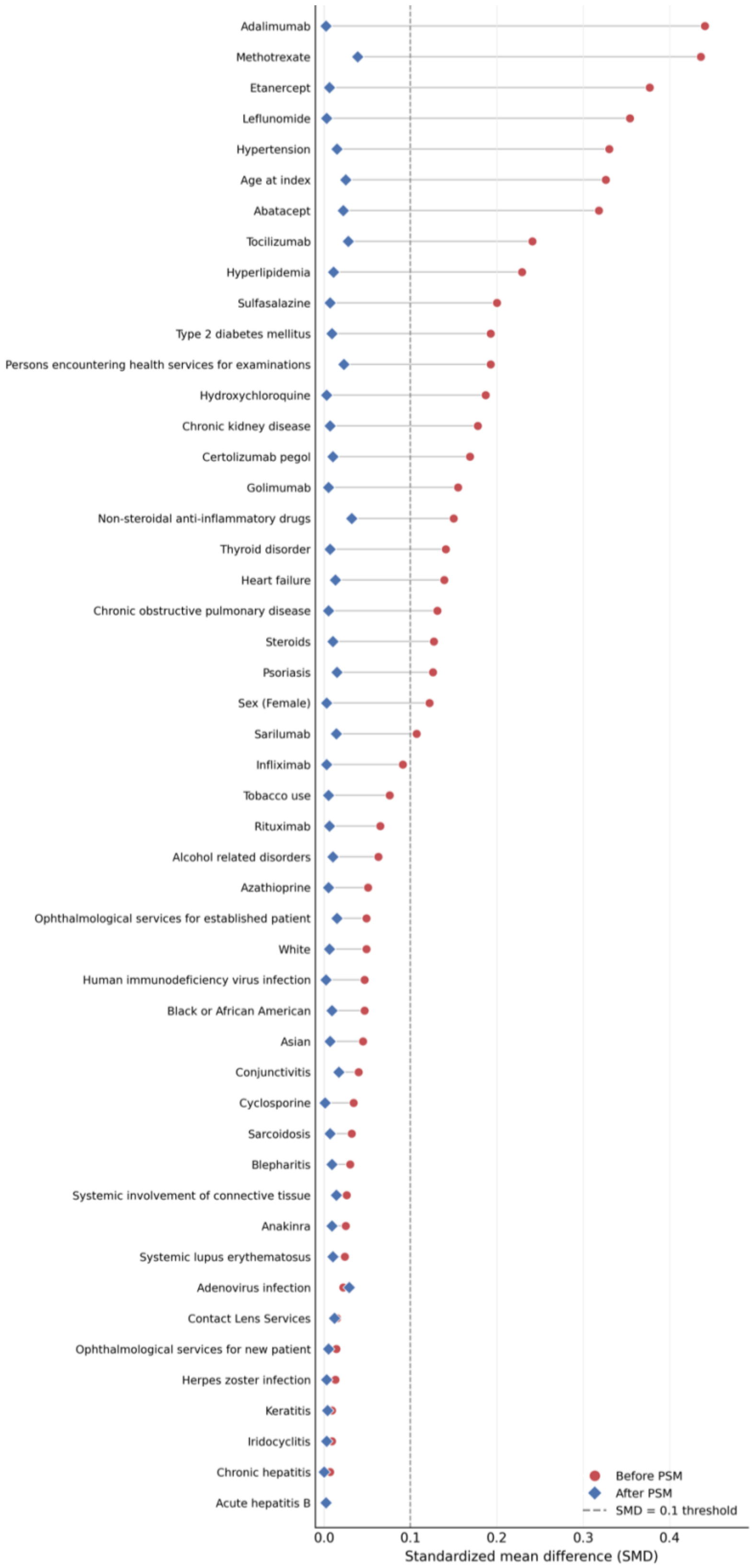

Love plot showing standardized mean differences for baseline covariates before and after propensity score matching between the JAK inhibitor cohort and the rituximab active-comparator cohort. After matching, all covariates were balanced with standardized mean differences below 0.1. Red circles represent covariate balance before PSM, and blue diamonds represent covariate balance after PSM.

Abbreviation: JAK, Janus kinase; PSM, propensity score matching; SMD, standardized mean difference.
